# Kidney injury in response to crystallization of calcium oxalate leads to rearrangement of the intrarenal T cell receptor delta immune repertoire

**DOI:** 10.1186/s12967-019-2022-0

**Published:** 2019-08-22

**Authors:** Chao Zhu, Qing Liang, Yaqun Liu, Deliang Kong, Jie Zhang, Hu Wang, Kejia Wang, Zhiyong Guo

**Affiliations:** 1 Department of Nephrology, Changhai Hospital, Second Military Medical University, Shanghai, 200433 China; 2 Department of Basic Medical Sciences, School of Medicine, Xiamen University, Xiamen, 361102 Fujian China; 3 Department of Rheumatology and Immunology, Changzheng Hospital, Second Military Medical University, Shanghai, 200003 China

**Keywords:** T cell receptor, Immune repertoire, γδT cell, Calcium oxalate, Kidney stone

## Abstract

**Background:**

Calcium oxalate (CaOx), the major constituent of most kidney stones, induces inflammatory infiltration and injures renal tubular cells. However, the role of γδT cells in CaOx-mediated kidney injury remains unclear. Therefore, this study investigated the distribution of intrarenal γδT cells and T cell receptor δ (TCRδ) immune repertoires in response to interactions with CaOx crystals.

**Methods:**

CaOx crystal mouse model was established by glyoxylate injection. Flow cytometer was used to analyze the expression of CD69 and IL-17 from intrarenal γδT cells. Furthermore, TCR immune repertoire sequencing (IR-Seq) was used to monitor the profile of the TCRδ immune repertoire.

**Results:**

Our results indicated that CaOx crystals lead to obvious increases in the expression and activation of intrarenal γδT cells. In TCRδ immune repertoire, the majority of V/J gene and V–J/V–D–J combination segments, barring individual exceptions, were similar between kidneys with CaOx formation and control kidneys. Impressively, high complementarity determining region 3 (CDR3) diversity was observed in response to CaOx crystal formation along with distinct CDR3 distribution and abundance.

**Conclusion:**

Our work suggests the presence of aberrant γδT cell activation and reconstitution of the TCRδ immune repertoire in response to CaOx crystal deposition.

## Background

Kidney stones, which primarily settle in the kidneys, are the most common disorder of the urinary tract, and their worldwide prevalence has increased over the decades [1]. The estimated prevalence of kidney stones in China is 650 per 10,000 people [2]. The most common renal stone component is CaOx, accounting for 65.9% of all stones. Hyperoxaluria is one of the primary risk factors for the occurrence of CaOx crystals, which may be influenced by a vegetarian diet and high carbohydrate intake [3]. Currently, it is widely accepted that kidney stones are a systemic disorder associated with an increased risk of chronic kidney diseases, renal cancer, metabolic disorder, renal failure, and cardiovascular diseases [4–7]. Kidney stones have become a serious problem in human health, and an appropriate prevention strategy is urgently needed.

γδT cells, consisting of a gamma (γ) and delta (δ) T cell receptor (TCR) chain, are primarily CD4^−^/CD8^−^ negative T cells. Although γδT cells normally account for 1–10% of circulating T lymphocytes, they constitute the major subset of resident T cells in the mucosa and organs [8, 9]. Unlike αβ T cells, γδT cells primarily sense early environmental stimuli, independent of an interaction with the major histocompatibility complex (MHC) on antigen-presenting cells (APCs), to mediate immunosurveillance and immunoregulation [10–12]. Therefore, γδT cells are characteristic of the adoptive immune compartment with innate-like response reactions. In inflammatory-mediated kidney injury, a variety of T cells (γδT cells, NKT cells, regulatory T cells) infiltrate the kidney, and the number and subset composition of infiltrating T cells varies among the different forms of kidney injury [13].

Emerging studies indicate that CaOx crystals boost the secretion of reactive oxygen species (ROS), proinflammatory cytokines, chemokines, and fibrotic factors, which aggravate renal interstitial inflammation in kidney stones [14–16]. In addition, there is evidence that these chemokines and cytokines subsequently enhance the recruitment of various immunocytes, including monocytes, macrophages, neutrophils, and T cells, to the CaOx-mediated inflammatory locale [17]. Nevertheless, the interaction between CaOx crystals and γδT cells and whether exposure to CaOx crystals induces alterations in γδT cell distribution and activation remain unknown.

Based on a preceding study, we deduced that IR-Seq represents a potential strategy for monitoring the immune microenvironment [18, 19]. In this study, we measured the quantity and activity of γδT cells in kidneys with CaOx formation, and TCR IR-Seq was used to monitor the expression pattern and clonality of the TCR repertoire in intrarenal γδT cells. Analysis of the TCRδ immune repertoires can improve our basic understanding of γδT cell immunology in renal stone disorders.

## Methods

### CaOx crystal mouse model

Wild-type male C57BL/6 mice (8–10 weeks old) were purchased from Daren Fortune Animal Technology Co., Ltd. (Qingdao, Shandong, China) and were maintained in the SPF barrier facility animal rooms under a controlled 12-h light/dark cycle at 20–25 °C with 55–56% relative humidity. All procedures were approved by the Second Military Medical University of the Medicine Institutional Animal Care and Use Committee. The CaOx crystal renal injury mouse model was established as previously described [20]. Briefly, mice were intraperitoneally (i.p.) injected with glyoxylate (Sigma-Aldrich, St. Louis, MO) at a dosage of 100 mg/kg or 0.9% saline once daily. After 7 days, kidneys were harvested. The left kidney was used for T cell isolation, and the right kidney was fixed in 4% paraformaldehyde for histological analysis.

### Histological analysis

Right kidney specimens were paraffin-embedded and sectioned at a thickness of 5 μm. Then, sections were deparaffinised, hydrated, and stained using a von Kossa Kit (Jiemei Gene, Shanghai, China) followed by subsequent Eosin counterstaining (Beyotime Institute of Biotechnology, Jiangsu, China). CaOx crystal deposition was assessed by 2 experienced pathologists using microscopy (Nikon Eclipse 50i; Nikon Corporation, Tokyo, Japan). Deposition scores were assigned as follows: (1) no deposition = 0; (2) deposition in the papillary tip = 1; (3) deposition in the corticomedullary junction = 2; (4) deposition in the cortex = 3.

Immunohistochemical staining was performed as previously described using a commercial immunohistochemical kit (SA1020, Boster, Hubei, China) [21]. Slides were incubated with primary antibodies against TCRγδ (GL3, 1:200, Abcam) overnight at 4 °C followed by incubation with secondary antibodies (Santa Cruz Biotechnology). Staining intensity was assessed using Image-Pro Plus 6.0 software (Media Cybernetics, MD, USA).

### T cell isolation and flow cytometry analysis

T cell isolation was performed as previously described. Briefly, a small incision was made in the portal vein, and a gauge needle was carefully inserted into the left ventricle of the heart to flush PBS through the cardiovascular system. Then, kidneys were mechanically disrupted into small pieces and transferred into digestion buffer [RPMI 1640 medium with 10 mg/mL Type 1 collagenase, 10 mg/mL DNAse, 10% foetal calf serum (FCS)] at 37 °C for 30 min. Next, 1 mL RPMI 1640 medium with 2% FCS was added to the suspension to stop digestion followed by filtration through a 100 mm nylon mesh to remove any remaining tissue fragments. When permeabilization was required, cells were incubated with 0.5% Triton X-100 (Solarbio, Beijing, China) for 10 min after fixation by 4% paraformaldehyde at room temperature. For γδT cell detection, cells were washed, and then stained using antibodies directed against mouse TCRγδ (Biolegend, Beijing, China), IL-17 (Biolegend) and CD69 (Biolegend) for 20 min at room temperature in the dark. BD Accuri C6 (BD Biosciences, Mountain View, CA, USA) was used to obtain and analyse the data.

### RNA extraction and TCR repertoires library preparation

Total RNA from γδT cells was extracted using the RNAprep Pure Cell/Bacteria Kit (Tiangen Biotech, Beijing, China). Quality and quantity of RNA were assessed using a NanoDrop spectrophotometer (Thermo Fisher Scientific, USA). Next, 200 ng RNA was utilized for reverse transcription using First-strand, and cDNA was synthesized using a Transcriptor First Strand cDNA Synthesis Kit (Roche Applied Science, Penzberg, Germany) according to the manufacturer’s protocol on a T100TM Thermal Cycler (Bio-Rad Inc., CA, USA).

### TCRδ repertoire preparation and IR-Seq

The TCRδ immune repertoire was amplified by two-round multiplex PCR using specific primers designed for TCRδ V and C regions according to the international ImMunoGene Tics information system (IMGT, http://www.imgt.org/). IR-Seq and data analysis were performed as previously described [19].

### Statistical analysis

Data are presented as the mean ± standard deviation. Statistical significance was determined using a 2-tailed, unpaired Student’s t-test or Mann–Whitney U test. Significance was accepted at *P *< 0.05. Heatmaps, volcano plots and principal component analysis (PCA) plots were generated using R software (version 3.5.1).

## Results

### CaOx-mediated kidney injury activates intrarenal γδT cells

**Fig. 1:**
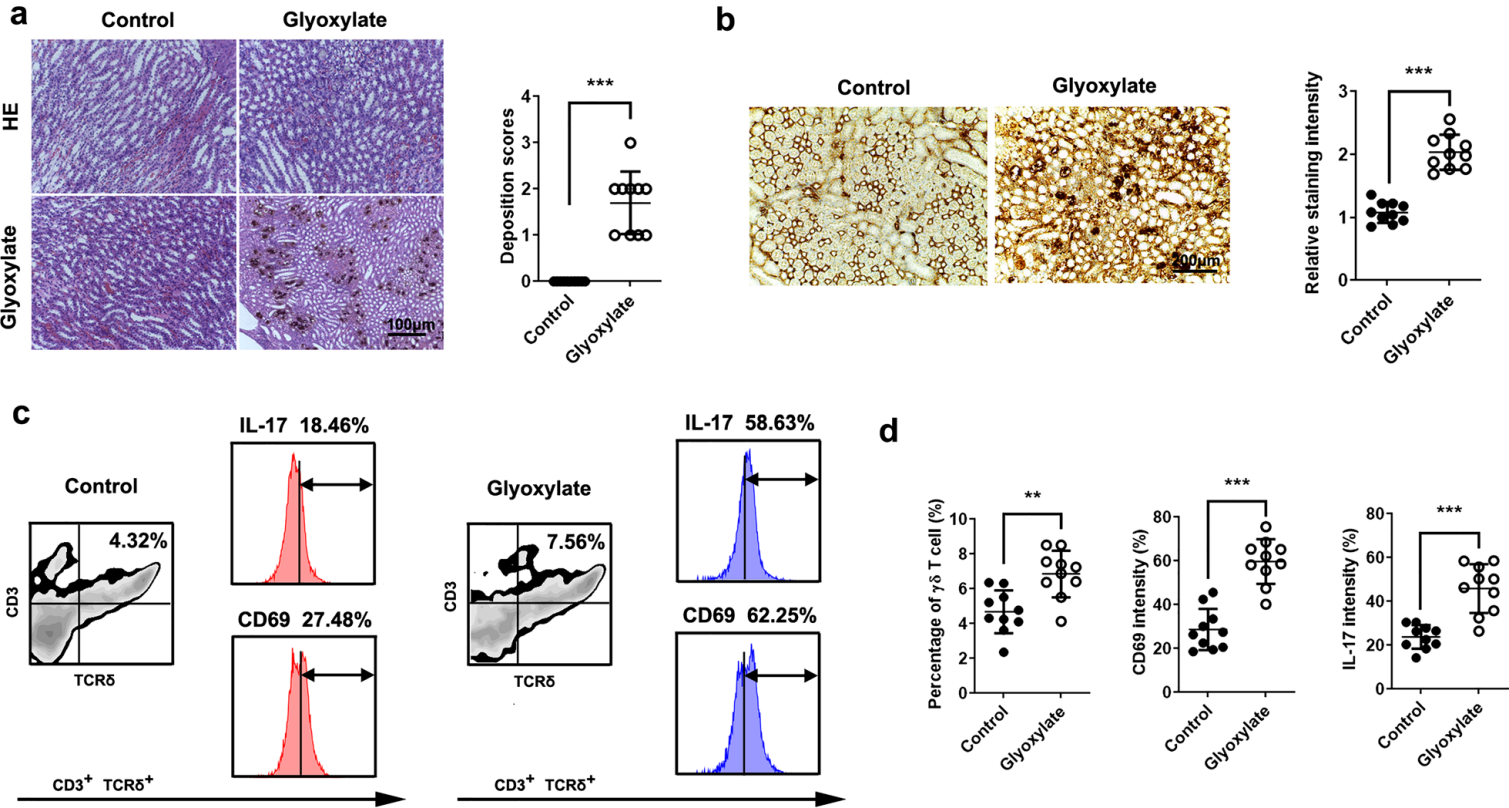
CaOx crystals activate γδT cells in the kidney. Ten mice were intraperitoneally injected with glyoxylate at 100 mg/kg or 0.9% saline once daily for 7 days. **a** HE and von Kossa staining was performed in mouse kidneys. Deposition scores were measured. **b** Immunohistochemical staining of TCRγδ in kidneys. The staining intensity of TCRγδ was calculated in 10 high power microscopic fields. **c** γδ T (CD3^+^, TCRγδ^+^) cells were gated and tested for the expression of CD69/IL-17 by flow cytometry. **d** The percentage of γδT cells and the expression of CD69 and IL-17 gated from γδT cells were quantified by flow cytometry (10 mice per group). *P < 0.05; **P < 0.01; ***P < 0.001

Haematoxylin and eosin (HE) and von Kossa staining revealed that glyoxylate induced the deposition of CaOx crystals in kidneys (Fig. 1a). To investigate the distribution of intrarenal γδT cells in response to CaOx, we labelled γδT cells by immunohistochemistry. The results indicated higher levels of intrarenal γδT cells in CaOx-mediated kidney injury compared to healthy kidneys (Fig. 1b). Furthermore, we examined the expression and activation of γδT cells by flow cytometry analysis. As expected, CaOx induced an obvious increase in the percentage of γδT cells, along with enhanced CD69 and IL-17 levels compared to controls, indicating significant activation of γδT cells in CaOx-induced kidneys (Fig. 1c, d).

### Profiling of V, D and J segment usage by γδT cells in CaOx-induced kidney injury

**Fig. 2:**
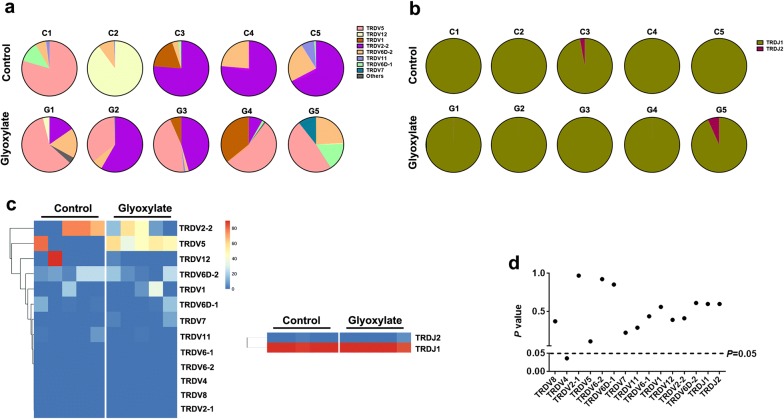
Distribution of V and J segments from the intrarenal TCRδ immune repertoire. IR-Seq of 10 TCRδ immune repertoire samples (5 glyoxylate and 5 control) from intrarenal γδT cells. **a**, **b** Frequency of V and J segments between glyoxylate and control groups. Colours indicate high frequency clones (each colour represents a clone); greys represent non-high frequency clones. **c** Heatmaps of clustering of V and J segment usage. **d** Comparison of V and J segment usage between the two groups (5 mice per group)

To explore the distribution and expression of γδT cells in response to CaOx crystal deposition, we assessed the intrarenal immune repertoire by IR-seq. This analysis yielded 1.26 × 10^7^ to 2.19 × 10^7^ reads per sample in raw data. V and J segments were identified using BLAST Plus software based on the IMGT database. Thirteen distinct V gene segments and 2 distinct J segments were observed from all samples, and there were few differences in V/J clonotypes between the two groups (Additional file 1: Dataset S1). Interestingly, the use of V and J segments was dominated by high-frequency clonotypes in both glyoxylate and control groups (Fig. 2a, b). Comparison of V and J frequencies revealed that the majority of V and J segment usage was similar (Fig. 2c). Only the frequency of TRDV4 was upregulated in the glyoxylate group compared to the control group (Fig. 2d).

**Fig. 3:**
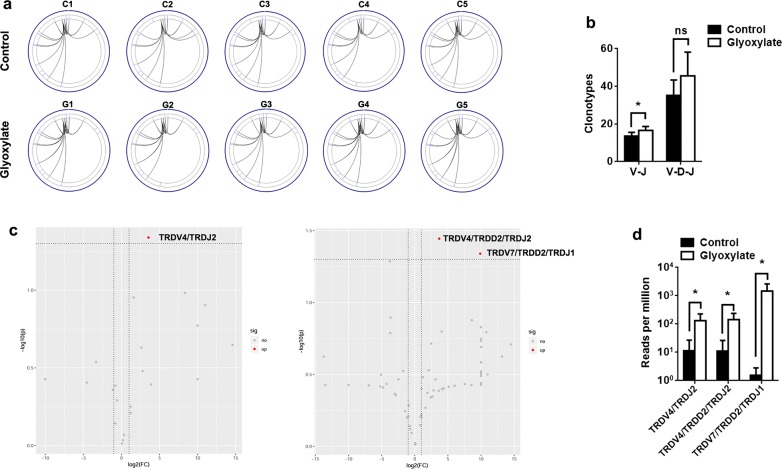
Patterns of V–J and V–D–J combination. **a** Circular plots representing V–J combinations from TCRδ loci. **b** Clonotypes of V–J and V–D–J combinations were identified. **c** Volcano plots representing V–J and V–D–J combinations between glyoxylate and control groups (red dots refer to upregulated combinations). **d** Comparison of V–J and V–D–J combinations with statistical significance testing (5 mice per group). *P < 0.05; **P < 0.01; ***P < 0.001

In addition, we determined the composition of paired V–J combinations and paired V–D–J combinations. There were a total of 22 V–J combinations and 93 V–D–J combinations (Additional file 2: Dataset S2). Of note, CaOx crystals led to diversified clonotypes of V–J and V–D–J combinations (Fig. 3a, b). Volcano plots were generated according to the frequency of V–J and V–D–J combinations (Fig. 3c). Similar to the use of V and J segments, the frequency of most V–J and V–D–J combinations was not different between the two groups. Only the frequencies of TRDV4/TRDJ2, TRDV4/TRDD2/TRDJ2 and TRDV7/TRDD2/TRDJ1 were increased in CaOx-induced damaged kidneys (Fig. 3d).

### CaOx crystals lead to a resetting of the TCRδ CDR3 clonotypes

**Fig. 4:**
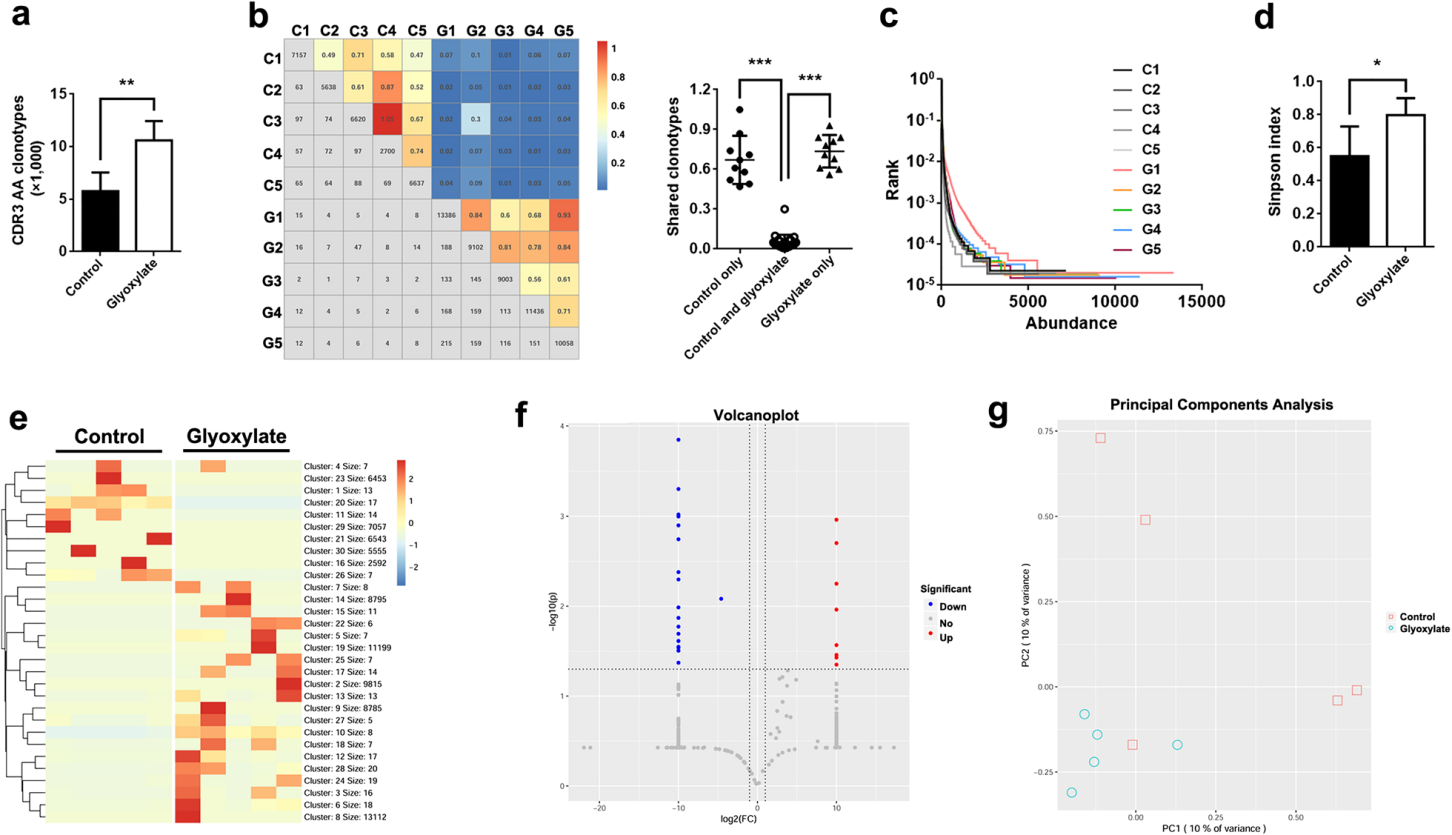
Reconstitution of CDR3 in response to CaOx crystals. **a** Comparison of total CDR3 clonotypes between glyoxylate and control groups. **b** Shared CDR3 clonotypes are shown as quantification (plot greys) and frequencies (plot colours). **c** Rank-abundance analysis of CDR3 clonotypes among different samples. **d** Simpson index in glyoxylate and control groups. **e** Heatmaps of clustering of V and J segment usage. **f** Volcano plots of CDR3 clonotypes in the two groups (red dots refer to upregulated CDR3 clonotypes; blue dots refer to downregulated CDR3 clonotypes). **g** PCA plot of glyoxylate and control group samples (5 mice per group). *P < 0.05; **P < 0.01; ***P < 0.001

CDR3, the most abundant region in the TCR repertoire, indicates the diversity of the TCR repertoire. In the current study, 79,307 distinct CDR3 clonotypes of TCRδ were identified (Additional file 3: Dataset S3). Of note, CaOx crystals led to remarkable increases in CDR3 TCRδ clonotypes (Fig. 4a). Interestingly, shared CDR3 clonotypes of samples among different groups were substantially lower than those in the same group, indicating reconstitution of TCRδ CDR3 clonotypes during CaOx crystal deposition (Fig. 4b). Moreover, rank-abundance analysis and the Simpson index suggested increased diversity of CDR3 clonotypes in CaOx-induced kidney injury (Fig. 4c, d).

A heatmap of CDR3 clonotype expression was generated by cluster analysis based on usage patterns. In accordance with CDR3 AA diversity, CaOx crystals diversified usage patterns of CDR3 clonotypes (Fig. 4e). Furthermore, we compared the frequency of CDR3 clonotypes between the two groups, identifying 19 downregulated CDR3 clonotypes and 8 upregulated CDR3 clonotypes (Table 1 and Fig. 4f). As shown by the PCA plot, the glyoxylate group exhibits centralized distribution, suggesting that the composition of CDR3 clonotypes in the glyoxylate group is clearly distinct from the control group (Fig. 4g). However, there were no significant differences observed in CDR3 AA usage or CDR3 AA length between the two groups (Additional file 4: Figures S1, S2).

**Table 1:** Analysis of CDR3 AA clonotypes in response to kidney injury by CaOx

CDR3 AA clonotypes	*P* value	Significant
ASGIWQISEGYELTDKL	0.0135	Down
AVYHCILRLIGGIRATDKL	0.0426	Down
AICGIHILLSEGYGTDKL	0.0243	Down
ALSELIVDKL	0.0083	Down
ASGYVAWGYRRVATDKL	0.0010	Down
ASGYMTSEGYELSDKL	0.0013	Down
ASGSMREDTDKL	0.0010	Down
ASGLIWPTEGYELTDKL	0.0001	Down
ALMEREGRRDTTDKL	0.0042	Down
ASQPSHSGTYLCGGKAGIRATDKL	0.0103	Down
ASGSTYRRDTYGATDKL	0.0313	Down
AVYHCILRLIWPIGGISTDKL	0.0244	Down
ASGLIWPTEGHELTDKL	0.0169	Down
ALMERARRDTHKL	0.0203	Down
ALSEVPSEGYAAPDKL	0.0283	Down
AVYHCILRGYGISEGSTDKL	0.0050	Down
ALMEQGGIRATDKL	0.0005	Down
ASQPSHSGTYLCGGGRGRYRRDTSSATDKL	0.0018	Down
ALWELAAEGYELSDKL	0.0290	Down
ATYYCGSDIGGSSWDTRQMSFGTGIEL	0.0348	Up
ARRAGGIRATDKL	0.0271	Up
ALMDRRVPATDKL	0.0056	Up
ASGAYIGGIRATDKL	0.0447	Up
ASGAYIGGIRTTDKL	0.0011	Up
AGMYYCGSDIGGSSWDTRQMF	0.0373	Up
ALSKLDMAYIGGIRATDKL	0.0109	Up
ALSELIGGIRATDKL	0.0020	Up

## Discussion

T cells play a critical role in immune-mediated kidney disorders, including glomerulonephritis, ischaemia–reperfusion injury, and renal fibrosis [22–24]. Accumulating evidence suggests that T cells act as helper cells for cytotoxic T cell production, for macrophage recruitment or as effector cells within kidneys by cytokine production, affecting immune function [25]. T cell dysfunction often causes acute and chronic inflammation that impairs renal function. Despite leukocyte infiltration after renal obstruction, far less is known about T cell distribution and function [24]. Nevertheless, there is clear evidence that γδT cells are essential for kidney injury [26]. Deficiencies in γδT cells cause minimal renal lesions and obviously reduced recruitment of other immunocytes [27].

γδT cells are involved in elimination of various pathogens, inflammation control and maintenance of tolerance via specific recognition using TCR [28]. Interestingly, γδT cells can also initiate adaptive effector functions without MHC restriction, suggesting direct presentation similar to that of antigen-presenting cells [8, 29]. In addition, the composition of γδT cells exhibits apparent imparity in diverse tissues. Therefore, the mechanisms of γδT cells in pathology are difficult to identify. Previous studies indicate that γδT cells mediate inflammatory cell infiltration (cytotoxic T cells and neutrophils) through IL-17A production, which contributes to pathogenesis and accelerates kidney injury [24, 27]. However, the role of γδT cells in CaOx-meditated renal injury is still unclear. A recent study suggested that γδT cells, especially IL-17A producing γδT cells, accumulate following unilateral ureteral obstruction-induced renal injury [24]. Consistent with previous studies, our results revealed elevated percentages and enhanced activation of γδT cells in kidneys in response to CaOx crystal deposition, implying a crucial role for γδT cells in CaOx crystal-mediated renal injury.

Emerging evidence demonstrates that endogenous and exogenous stimuli lead to TCR immune repertoire rearrangement, facilitating antigen recognition of TCR [30–32]. Indeed, enhanced activation of γδT cells can be attributed to CaOx crystals and CaOx crystal-mediated renal injury. Nevertheless, the profile of the TCRδ immune repertoire during the process of CaOx crystal deposition is not clear. Our preceding studies propose that TCR acts as a “commander” to monitor the immune microenvironment and allocate immunocytes for immune elimination and immune repair [33]. Currently, TCR is understood to be one of the pathophysiological mediators in kidney ischaemia–reperfusion injury, and identification of TCR function in the kidney could also be conducive to identifying new therapies for renal injury [23]. Therefore, identifying and tracking TCR immune repertoires provides a novel strategy for understanding the mechanism of T cells in renal injury.

In this study, characteristics of V, D, J and CDR3 usage patterns were measured using IR-seq. Of note, a more centralized distribution of V and J segments was identified in both glyoxylate and control groups. Our results coincide with previous findings that the TCR V region repertoire in particular is highly tissue specific, with 75% of peripheral blood γδT cells expressing TRDV2 [34]. Interestingly, the proportion of γδT cells expressing TRDV5 increased in response to CaOx crystal deposition but did not reach statistical significance. Although the majority of V, J, V–J and V–D–J combination usage were similar, CaOx crystal deposition induced diversification of the clonotypes of V–J and V–D–J combinations. Moreover, obviously elevated CDR3 clonotypes and variance were observed in the glyoxylate group, suggesting that CaOx crystal deposition leads to enhanced TCRβ diversity and VDJ recomposition. These data support that TCR immune repertoire rearrangement occurs to facilitate the recognition of renal autoantigens, which induces γδT cell activation and kidney injury. Meanwhile, chemokines and cytokines recruit other immunocytes synergistically with γδT cells to promote renal inflammation.

Indeed, the TCRδ immune repertoire exhibits traits of the immune microenvironment in kidneys in response to CaOx crystal deposition, thus improving our understanding of the role of γδT cells in renal injury in response to CaOx crystal deposition. Interestingly, our ongoing work also finds the rearrangement of the intrarenal TCRβ immune repertoire during renal ischemia reperfusion injury, which demonstrates that TCR reconstitution may be a common occurrence in kidney diseases. However, the relationship between the TCR reconstitution and TCR signalling pathway or biological effect (including T cell activation) are still not clear. Unfortunately, the biological effect of TCRδ immune repertoire reconstitution is still unknown. In future studies, the mechanism and signalling pathways that associate with the TCRδ immune repertoire will be assessed.

## Conclusion

The present study demonstrates that CaOx crystal deposition causes γδT cell accumulation and activation. Further IR-seq of TCRδ elucidated anomalous TCRδ immune repertoire diversity. Transformation of the TCR immune repertoire might be therapeutically exploited in kidney injury in the future.

## Supplementary information

**Additional file 1.** Comparison of TRDV and TRDJ usage.

**Additional file 2.** Comparison of V–J and V–D–J usage.

**Additional file 3.** Comparison of CDR3 AA usage.

**Additional file 4: Figure S1.** Distribution of CDR3 AA length in the TCRδ immune repertoire between glyoxylate and control groups. **Figure S2.** Comparison of the fraction of CDR3 AA between glyoxylate and control groups.

## Data Availability

The datasets supporting the conclusions of this article are included within the article.

## Abbreviations

CaOx: calcium oxalate
TCR: T cell receptor
IR-Seq: immune repertoire sequencing
CDR3: complementary determined regions 3
MHC: major histocompatibility complex
APCs: antigen-presenting cells
ROS: reactive oxygen species
IMGT: ImMunoGene Tics information system
HE: haematoxylin and eosin
