# Supplementary material for: Kidney injury in response to crystallization of calcium oxalate leads to rearrangement of the intrarenal T cell receptor delta immune repertoire
Source: J Transl Med. 2019 Aug 22;17:278. doi: 10.1186/s12967-019-2022-0 (PMC6704580; doi:10.1186/s12967-019-2022-0)
Supplement: Supplementary file 4 — Additional file 4: Figure S1. Distribution of CDR3 AA length in the TCRδ immune repertoire between glyoxylate and control groups. Figure S2. Comparison of the fraction of CDR3 AA between glyoxylate and control groups. [file 12967_2019_2022_MOESM4_ESM.doc]

**Kidney injury in response to crystallization of calcium oxalate leads to rearrangement of the intrarenal T cell receptor delta immune repertoire**

Chao Zhu^1*^, Qing Liang^2*^, Yaqun Liu^3*^, Deliang Kong^1^, Jie Zhang^1^, Hu Wang^1^, Kejia Wang^2^, Zhiyong Guo^1^

^1^ Department of Nephrology, Changhai Hospital, Second Military Medical University, Shanghai, China 200433. ^2^ Department of Basic Medical Sciences, School of Medicine, Xiamen University, Xiamen, Fujian, China 361102. ^3^ Department of Rheumatology and Immunology, Changzheng Hospital, Second Military Medical University, Shanghai, China 200003.

^*^Chao Zhu, Qing Liang and Yaqun Liu contributed equally to this work.

**Correspondence**

Kejia Wang (Email: wangkejia@xmu.edu.cn) and Zhiyong Guo (Email: drguozhiyong@outlook.com)

**Figure S1** Distribution of CDR3 AA length in the TCRδ immune repertoire between glyoxylate and control groups.

**Figure S2** Comparison of the fraction of CDR3 AA between glyoxylate and control groups.
